# Prospective assessment of intraoperative renal perfusion with transit time flow measurements (TTFM) in deceased and living donor kidney transplantation

**DOI:** 10.1007/s13304-025-02344-5

**Published:** 2025-08-25

**Authors:** Roberto I. Troisi, Gianluca Rompianesi, Silvia Campanile, Xenios Eleftheriades, Fabiana Rubba, Gianluca Cassese, Marcello Caggiano, Giuseppe De Simone, Mariano C. Giglio, Rosa Carrano, Umberto M. Bracale, Roberto Montalti

**Affiliations:** 1 Division of HPB, Minimally Invasive and Robotic Surgery, Renal Transplant Service, Department of Clinical Medicine and Surgery, Federico II University, Via Pansini 5, 80131 Naples, Italy; 2 Department of Public Health, Federico II University, Naples, Italy; 3 Department of Anesthesiology and Intensive Care, Federico II University, Naples, Italy; 4 Unit of Nephrology and Transplantation, Federico II University Hospital, Naples, Italy; 5 Vascular Surgery Unit, Federico II University Hospital, Naples, Italy

**Keywords:** Kidney transplant, Flow measurements, Vascular complications, Delayed graft function

## Abstract

Optimal graft perfusion is key to achieving satisfactory post-transplant function. The possibility of evaluating vascular flows can lead to the early identification of vascular complications and reflect graft quality and outcome. From 1, 2022 to 1, 2024, transit time flow measurements (TTFM) were prospectively recorded in 75 consecutive kidney transplants (KTx) and analyzed alongside donor, recipient, transplant, and outcome data. Correct measurements were obtained in all cases. Patients receiving living-donor transplants showed higher arterial TTFM (397 (251–532) vs. 295 (167–382) ml/min, *p* = 0.010) but similar venous TTFM (*p* = 0.512). Arterial TTFM presented an inverse correlation with donor BMI (*r* = − 0.241, 95% CI − 0.449–0.008, *p* = 0.037). Two patients (2.6%) with severely reduced TTFM (< 50 ml/min) developed intraoperative vascular complications and underwent immediate treatment. Patients experiencing delayed graft function (DGF) presented lower arterial and venous TTFM (200 (119–298) vs. 341 (267–448) ml/min, *p* < 0.001 and 222 (170–391) vs. 369 (272–456) ml/min, *p* = 0.015), respectively. In patients with higher arterial TTFM, the serum creatinine levels showed a faster decrease (*r* = − 0.493, 95% CI − 0.652–0.293, *p* < 0.001). Arterial TTFM (OR: 0.993 (0.989–0.998), *p* = 0.004) and donor arterial hypertension (OR: 9.292 (2.337–36.935), *p* = 0.002) resulted in independent risk factors for DGF development at the multivariable logistic regression analysis. The identified arterial cutoff for better outcomes was 310 ml/min (AUROC 0.765). The intraoperative TTFM evaluation in KTx was safe and effective in the early recognition of vascular complications. Arterial TTFM reflect graft quality, with lower flows (< 310 ml/min) correlating with slower post-transplant serum creatinine decrement and representing an independent risk factor for DGF development.

## Introduction

Kidney transplantation (KTx) is the treatment of choice for patients with end-stage chronic kidney disease, providing significant survival and quality of life benefits [1]. Additionally, KTx has proven to be cost-effective not only with organs from living or standard-criteria donors, but also with expanded criteria organs [2]. With expected 1-year graft survival rates exceeding 95% [3], the proportion of patients undergoing KTx and experiencing early graft loss is less than 5% but is known to lead to inferior long-term survival outcomes [4]. The most frequent causes of early graft losses are vascular complications, mainly arterial and venous thrombosis [5]. Optimal renal perfusion is essential following KTx to re-establish prompt and adequate function and avoid transplant failure. However, several donor, graft, and recipient factors need to be considered as potential determinants. Organ perfusion impairment can start in situ in deceased donors even before organ retrieval, as brain death leads to a sympathetic storm causing raised systemic vascular resistances and visceral ischemia, usually followed by a sudden loss of sympathetic tone, provoking severe vasodilation. This, combined with the active inflammatory response, leads to endothelial damage and ischemia–reperfusion injury (IRI) [6, 7]. In donors after circulatory death (DCD), the additional hypoperfusion and warm ischemia time further increase the risk of delayed graft function (DGF) [8]. The unavoidable ischemia during the organ donation and retrieval process is also an established cause of renal vascular rarefaction and microvasculature obstruction [9]. A strategy to hemodynamically stimulate the kidney graft vasculature to prevent endothelial damage is machine perfusion preservation, which has been proven to result in better and immediate function and superior graft survival [10, 11].

Despite the advances achieved in the last decades in the field of transplant immunology, episodes of graft rejection can negatively impact short- and long-term survival outcomes. In particular, hyperacute rejection, caused by preformed donor-specific antibodies, represents an infrequent event but has the potential to lead to early graft loss [12].

Another factor that can negatively impact graft perfusion and subsequent function is the hemodynamic status of the recipient. A key role in maintaining an optimal hemodynamic balance is played by the anesthetic team in the perioperative period. This can be subject to significant imbalances in patients with chronic renal disease undergoing dialysis treatment and the intraoperative use of catecholamine vasopressors to maintain an adequate blood pressure [10, 13, 14]. During vessel isolation, the iliac artery can often experience acute vascular spasm, which, even if transitory, can lead to thrombosis and distal ischemia, affecting the graft and the limb [15].

Considering all the above, the accurate intraoperative evaluation of renal graft vascular flows in a simple, reproducible, and non-invasive manner is highly relevant for the early identification of inadequate renal perfusion.

In this study, we retrospectively analyzed prospectively recorded intraoperative post-implantation renal flow direct measurements and explored any possible correlation with graft outcomes in living donor and deceased donors after brain death KTx.

## Materials and methods

From January 2022 to January 2024, 75 consecutive KTx were performed at Federico II University Hospital Transplant Centre (Naples, Italy) and prospectively evaluated with the transit time flow measurement (TTFM) probes (MiraQ™ Medistim ASA, Oslo-Norway) and donor, recipient, transplant, TTFM, and outcome data in terms of DGF, PNF, graft and patient survival, post-KTx renal function were collected in a prospective database and analyzed. The local Ethics Committee approval was requested and obtained (34/2024, n. 2/24).

### Surgical procedures

All deceased donor kidney retrievals were performed according to the standard rapid technique, with an aortic patch included with the renal arteries and a segment of inferior vena cava (IVC) with the right renal vein. The organ perfusion was performed in situ and at the back-table with Celsior solution (Institut Georges Lopez, 69,380 Lissieu–France). An IVC elongation on the right graft renal veins was performed in cases of length discrepancy with the renal artery. Multiple arteries were maintained on the same patch and implanted with a single anastomosis, when feasible, or separately. When performed, the graft machine perfusion was performed in a hypothermic setting (HMP), with the Waves IGL® Transportable Perfusion System.

All living donor nephrectomies were performed with a fully robotic-assisted technique (Da Vinci Xi platform, Intuitive Sunnyvale CA-USA), dividing the renal vessels with the Signia™ stapler with small-diameter 7 mm vascular cartridges in order to achieve the maximum possible length. The vascular stumps were further reinforced with Hem-O-Lock clips (Teleflex Medical Europe, Dublin Ireland) clips were additionally securing the arterial stapler line. The grafts were extracted through a mini Pfannenstiel incision in an organ retrieval bag.

Kidney implantations were performed through a Rutherford-Morrison incision, with end-to-side vascular anastomoses following segmental external iliac vein and artery clamping.

The immunosuppressive therapy consisted of a triple therapy based on intraoperative and postoperative day 4 induction with anti-IL2-R monoclonal antibody (basiliximab), steroids, tacrolimus, and enteric-coated mycophenolate sodium.

### Intraoperative transit time flow measurements (TTFM)

**Fig. 1:**
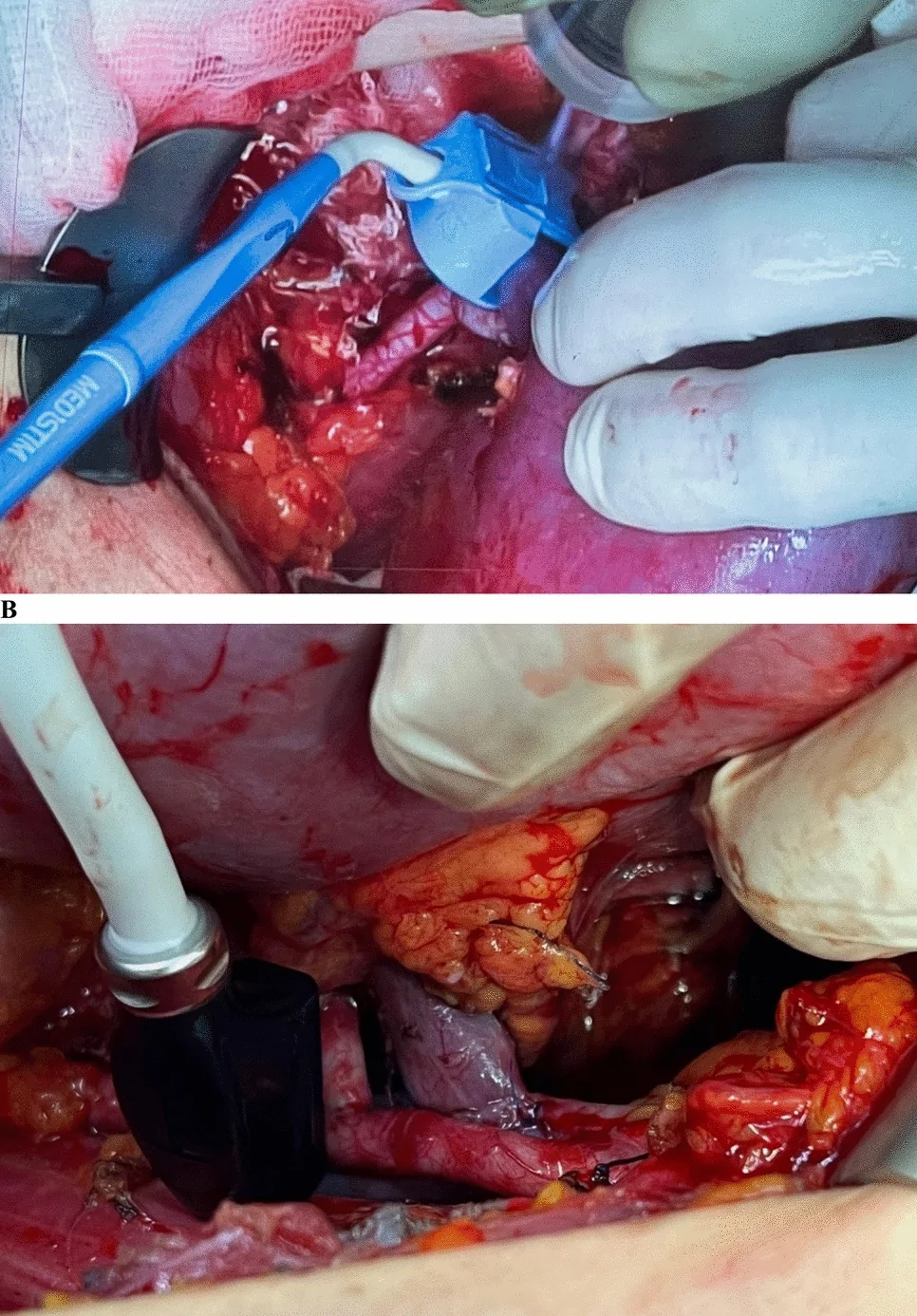
**A, B** Intraoperative post-reperfusion transit time flow measurements of the renal vein (TTFM, 2 A) and colour-doppler high-frequency ultrasound imaging evaluation of the renal artery and the anastomotic site (HFUS, 2B)

**Fig. 2:**
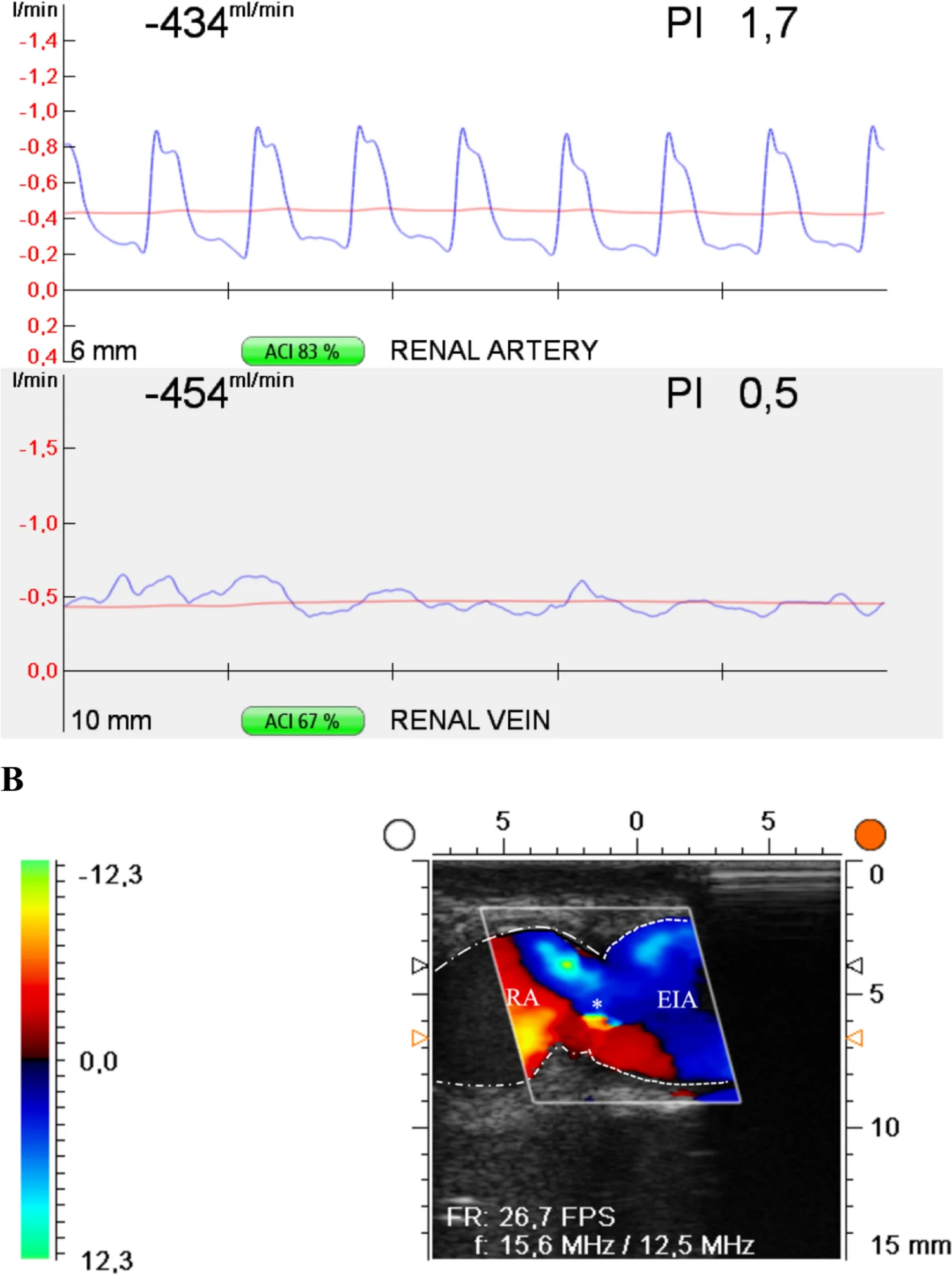
**A, B** Optimal post-reperfusion renal artery and vein transit time flow measurements (TTFM, 2 A) and colour-doppler high-frequency ultrasound imaging evaluation (HFUS, 2B). PI, pulsatility index; ACI, acoustic coupling index; RA, renal artery; EIA, external iliac artery, * (site of anastomosis)

After graft reperfusion, TTFM were obtained on the renal artery and vein with the MiraQ™ Vascular system by positioning the adequate size (from 3 to 12 mm) vascular TTFM probes (Fig. 1A) on the renal artery and vein and measuring the flow (ml/min) and the pulsatility index (PI) [16]. TTFM were performed in all vessels in case of multiple arterial or venous branches, unless in the presence of very small (< 3 mm) or spasmed vessels where further manipulation was avoided. A pre-anastomotic and post-reperfusion arterial anastomosis evaluation with high-frequency ultrasound imaging (HFUS) was performed in order to obtain a detailed vascular morphological assessment (Figs. 1B and 2).

### Statistical analysis

Descriptive statistics for all collected variables have been performed. The distribution characteristic of the measured data was analyzed with the Shapiro–Wilk test and the homogeneity test of variances. Normally distributed quantitative variables have been reported as mean values and standard deviation (SD) and compared using the two-sided Student’s t-test; otherwise, they have been reported as median and interquartile range and compared using the Mann–Whitney test. Qualitative variables are expressed as counts and percentages and compared using the Chi-Squared test with Yates’ correction or the Fisher’s exact test. Uni- and multivariable logistic regression analyses with backward stepwise selection have been performed in order to identify independent risk factors of DGF occurrence, including patient and donor demographics, graft, transplant, and TTFM characteristics. All variables that resulted statistically significant in the univariable analyses have been included in the multivariable model. Potential multicollinearity between variables has been checked by Spearman’s rank correlation coefficient. Patient overall survival and graft survival were evaluated using Kaplan–Meier curves. Receiver operating characteristic (ROC) curve analyses with Youden Index were used to calculate the optimal cut-off values for the continuous variables and the area under the receiver operator characteristic (AUROC) evaluated.

*p*-Values < 0.05 have been considered statistically significant. All tests have been two-sided. The data analyses have been performed with SPSS (version 28, IBM, New York, NY, USA).

## Results

Seventy-five consecutive KTx were included in the analysis; 16 of them were living-donor (LD) KTx (21.3%) and the remaining 59 (78.7%) were from deceased donors (DD) after brain death (DBD), including 1 (1.3%) dual-graft transplant. In 7 cases (9.3%) the DBD grafts underwent hypothermic machine perfusion (MP). The average recipient age was 45.5 ± 10.2 years, and the most frequent cause of chronic kidney disease was autosomal dominant polycystic kidney disease (ADPKD) in 28% of cases, with 12 (16%) pre-emptive KTx and 6 (8%) patients who received a previous KTx; the average kidney donor profile index (KDPI) was 42.1 ± 16.4. The full donor, recipient, and transplant characteristics are reported in Table 1.

**Table 1:** Demographic, baseline, and donor characteristics of the included patients

		Whole Cohort 75 patients	CS-DD KTx 52 patients (69.3%)	MP-DD KTx 7 patients (9.3%)	LD KTx 16 patients (21.3%)
Age, years	average ± SD	45.5 ± 10.2	45.7 ± 10.1	50.6 ± 9.9	42.6 ± 10.2
Gender, M	N (%)	35 (46.7)	25 (48.1)	2 (28.6)	8 (50)
BMI, kg/m^2^	average ± SD	24.9 ± 4.5	25.2 ± 4.6	24.7 ± 4.6	24.23 ± 4.5
CKD etiology					
ADPKD	N (%)	20 (26.7)	12 (23.1)	2 (28.6)	6 (37.5)
Urological		7 (9.3)	5 (9.6)	0	2 (12.5)
Hypertensive nephropathy		6 (8)	5 (9.6)	0	1 (6.2)
Glomerulonephritis		4 (5.3)	3 (5.8)	1 (14.3)	0
FSGS		3 (4)	2 (3.8)	0	1 (6.2)
IgA nephropathy		3 (4)	2 (3.8)	0	1 (6.2)
Congenital hypoplasia		3 (4)	1 (1.9)	0	2 (12.5)
Lupus		3 (4)	2 (3.8)	0	1 (6.2)
Failed		3 (4)	2. (3.8)	1 (14.3)	0
Previous KTx					
Other		9 (12)	7 (13.5)	2 (28.6)	0
NDD		14 (18.7)	11 (21.2)	1 (14.3)	2 (12.5)
Pre-KTx dialysis					
HD	N (%)	61 (81.3)	46 (88.5)	6 (85.7)	9 (56.3)
PD		2 (2.7)	1 (1.9)	0	1 (6.2)
Pre-emptive		12 (16)	5 (9.6)	1 (14.3)	6 (37.5)
Previous KTx	N (%)	6 (8)	5 (9.6)	1 (14.3)	0
Donor age, years	average ± SD	45.5 ± 10.2	45.7 ± 10.1	50.6 ± 9.9	42.6 ± 10.2
Donor BMI	average ± SD	25.2 ± 4.2	25.4 ± 4.2	26.8 ± 4.2	23.6 ± 4.2
Donor GFR, ml/min	average ± SD	106.1 ± 44.2	116.1 ± 44.4	68.6 ± 45.3	91 ± 44
Donor hypertension	N (%)	17 (22.7)	20 (38.5)	3 (42.9)	0
Donor diabetes	N (%)	1 (1.3)	1 (1.9)	0	0
KDPI	average ± SD	42.1 ± 16.4	42.3 ± 16.4	59.0 ± 15.0	34.4 ± 10.8
Ischaemia time, min					
CIT	Median (IQR)	506 (315–692)	544 (414–716)	873 (646–1094)	91 (64–106)
WIT		34 (28–45)	36 (30–53)	34 (26–44)	28 (23–34)

KTx, kidney transplant; CS-DD, cold storage preserved deceased donors; MP-DD, machine perfusion preserved deceased donors; LD, living donor; SD, standard deviation; M, male; BMI, body mass index; CKD, chronic kidney disease; ADPKD, adult polycystic kidney disease; FSGS, focal segmental glomerulosclerosis; HD, hemodialysis; PD, peritoneal dialysis; GFR, glomerular filtration rate; KDPI, kidney donor profile index; CIT, cold ischemia time; WIT, warm ischemia time; IQR, interquartile range

### Intraoperative transit time flow measurements (TTFM)

**Fig. 3:**
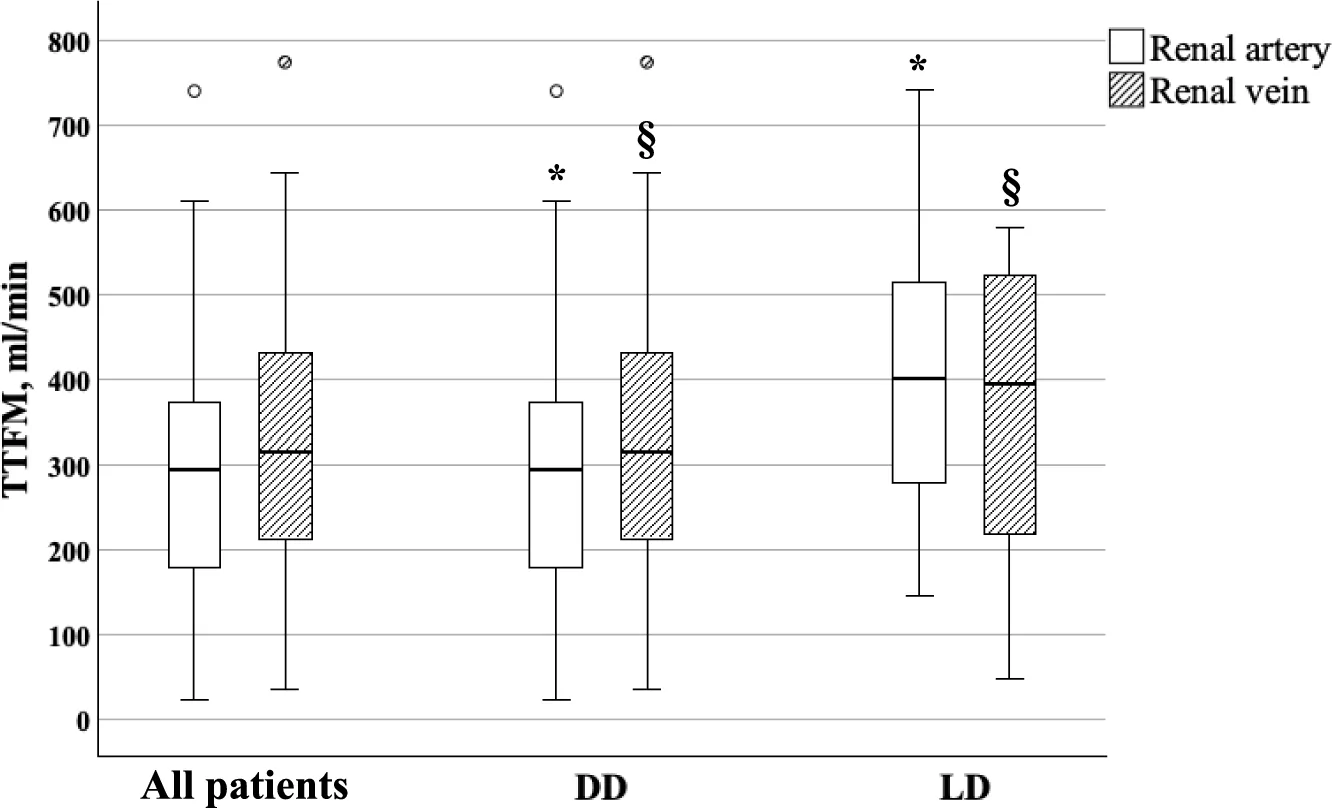
Renal artery and vein transit time flow measurements (TTFM) according to the donor type. LD, living donors; DD, deceased donors. * Renal artery TTFM in kidney transplants following living versus deceased donor: *p* = 0.010. § Renal vein TTFM in kidney transplants following living versus deceased donor: *p* = 0.512

In all patients the intraoperative TTFM resulted successful and did not lead to any complication or adverse event. In the whole cohort, the median measured renal artery flow was 300 (200–428) ml/min with a PI of 1.4 (1.0–2.0), and the median renal vein flow was 319 (210–432) ml/min with a PI of 0.7 (0.4–1.1). Patients receiving a LD KTx showed higher renal artery TTFM (397 (251–532) vs. 295 (167–382) ml/min, *p* = 0.010), with similar renal vein TTFM (395 (195–531) vs. 316 (211–430) ml/min, *p* = 0.512). KTx from standard criteria deceased donors (SCD) presented renal artery TTFM comparable to ECD (295 (165–395) vs. 268 (167–336) ml/min, *p* = 0.561) with higher renal vein TTFM (323 (228–453) vs. 259 (155–378) ml/min, *p* = 0.040). Among DD recipients, grafts transplanted following static cold storage (SCS) presented higher, although not statistically significant, TTFM compared to the KTx following HMP (renal artery: 297 (192–409) vs. 193 (140–269) ml/min, *p* = 0.131, renal vein: 323 (215–431) ml/min vs. 302 (140–430) ml/min, *p* = 0.401). The full intraoperative TTFM are reported in Table 2 and Fig. 3. The arterial TTFM appeared to be inversely correlated with the donor BMI (*r* = − 0.241, 95% CI − 0.449 to − 0.008, *p* = 0.037), with lower flows observed in patients receiving a KTx from donors with higher BMI values.

**Table 2:** Intraoperative characteristics and vascular flow measurements of the included patients

		Whole Cohort 75 patients	CS-DD KTx 52 patients (69.3%)	MP-DD KTx 7 patients (9.3%)	LD KTx 16 patients (21.3%)
Number of renal arteries					
Single	N (%)	60 (80)	39 (75)	6 (85.7)	15 (93.7)
Multiple		15. (20)	13 (25)	1 (14.3)	1 (6.3)
Number of renal anastomoses					
Single	N (%)	72 (96)	50 (96.2)	6 (85.7)	16 (100)
Multiple		3 (4)	2 (3.8)	1 (14.3)	0
Number of renal veins					
Single	N (%)	72 (96)	51 (98.1)	6 (85.7)	15 (93.7)
multiple		3 (4)	1 (1.9)	1 (14.3)	1 (6.3)
Number of venous anastomoses					
Single	N (%)	73 (97.3)	51 (98.1)	6 (85.7)	16 (100)
Multiple		2 (2.7)	1 (1.9)	1 (14.3)	0
Number of ureteric anastomoses					
1	N (%)	72 (96)	51 (98.1)1 (1.9)	5 (71.4)	16 (100)
2		3 (4)	1 (1.9)	2 (28.6)	0
Renal artery flow, ml/min	Median (IQR)	300 (200–428)	297 (192–409)	193 (140–269)	397 (251–532)
Renal artery PI	Median (IQR)	1.4 (1.0–2.0)	1.5 (1.1–2.0)	2.0 (1.5–2.5)	0.9 (0.8–1.2)
Renal vein flow, ml/min	Median (IQR)	319 (210–432)	323 (215–431)	302 (140–430)	395 (195–531)
Renal vein PI	Median (IQR)	0.7 (0.4–1.1)	0.6 (0.4–1.1)	0.8 (0.7–1.4)	0.7 (0.4–1.1)

KTx, kidney transplant; CS-DD, cold storage preserved deceased donors; MP-DD, machine perfusion preserved deceased donors; LD, living donor; IQR, interquartile range; PI, pulsatility index

### Outcomes, patient and graft survival

**Fig. 4:**
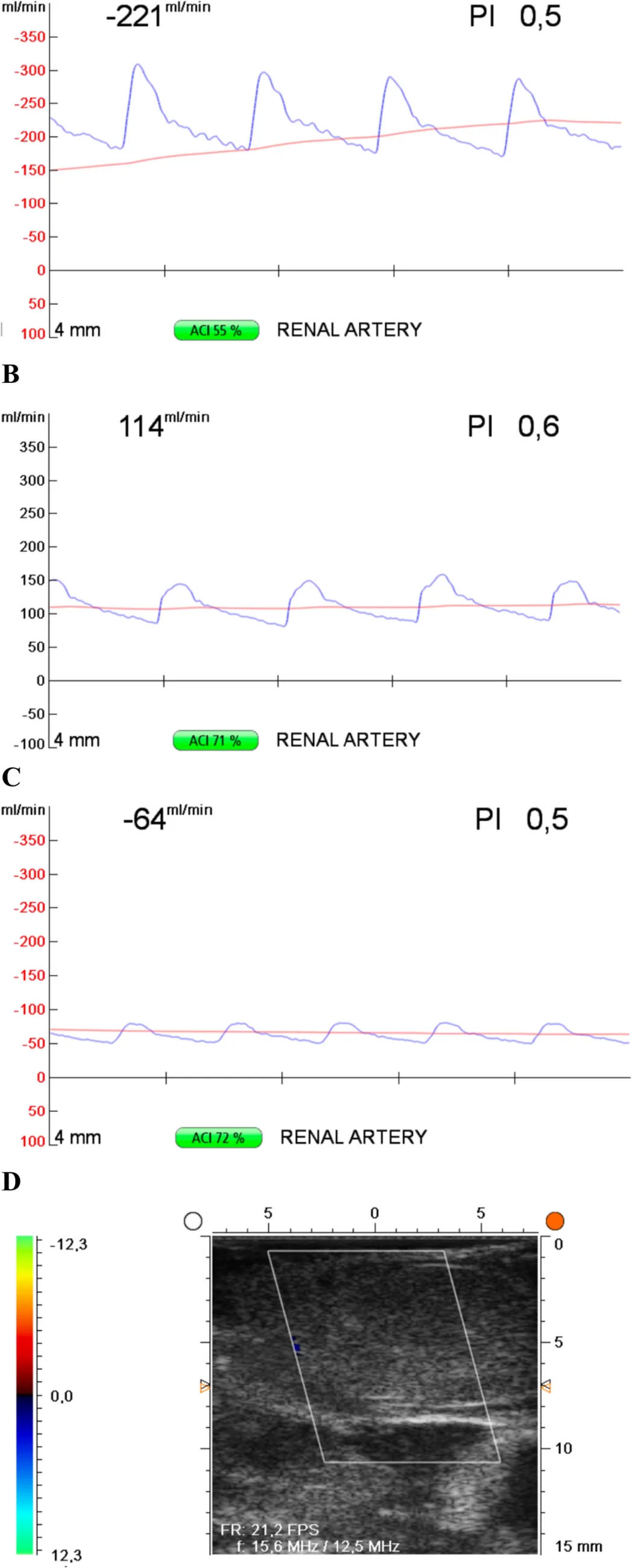
Progressively decreasing renal artery transit time flow measurements (TTFM) in a patient presenting post-reperfusion external iliac artery dissection and renal artery thrombosis. Renal artery TTFM at 5 (4A), 10 (4B) and 15 min (4C) from graft reperfusion. The peaks of the Doppler waves appear to be flattened and are associated with a significantly decreased arterial flow, due to the thrombosed post-anastomotic renal artery. Absent renal artery intraluminal flow at the color-Doppler high-frequency ultrasound imaging evaluation (4D). PI, pulsatility index; ACI, acoustic coupling index

**Fig. 5:**
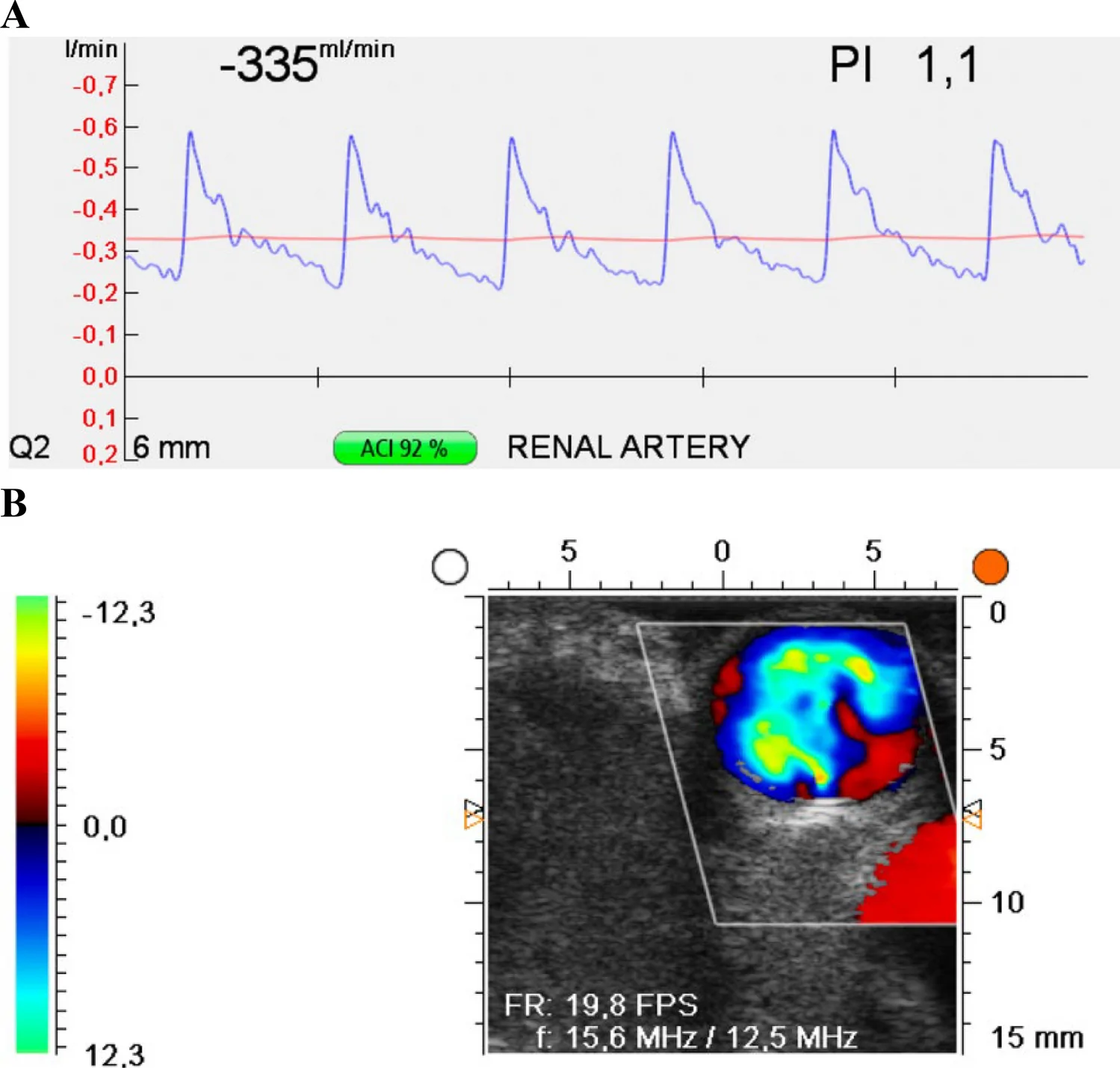
Optimal renal artery transit time flow measurements (TTFM) after contralateral graft re-implantation following external iliac artery dissection and renal artery thrombosis (5A). Satisfactory colour-doppler high-frequency ultrasound imaging evaluation of the post-anastomotic renal artery (5B). PI, pulsatility index; ACI, acoustic coupling index

All patients were followed up for an average of 13.5 ± 7.2 months after the KTx. Two patients (2.7%) died during the follow-up 6 and 25 months after the transplant due to cardiorespiratory insufficiency and sepsis. Overall patient and graft survival at 1 year were 98.6% and 91.6%, respectively. LD recipients showed better, although not statistically significant, patient and graft survival (both 100%) compared to deceased donor recipients (98.2% and 89.4%, respectively). At the end of the follow up, the average glomerular filtration rate (GFR) was 106 ± 44 ml/min, with 27 patients (36%) having experienced DGF, 1 following LD KTx (6.2%) and 26 DD KTx (44.1%), *p* = 0.004. In five patients (6.7%) a graft loss within 6 months of KTx was observed. Causes of graft loss were an early graft venous thrombosis requiring transplant nephrectomy, sepsis, hemolytic-uremic syndrome, and in cases where it was not possible to determine a diagnosis despite the kidney biopsies. The patient experiencing a graft loss due to renal vein thrombosis on postoperative day 1 presented very low arterial and venous intraoperative TTFM (23 and 35 ml/min, respectively). In one patient receiving a living donor graft (1.3%), immediate intraoperative unsatisfactory arterial TTFM and the HFUS evaluation (Fig. 4) highlighted inadequate graft perfusion due to external iliac artery peri-anastomotic dissection, mandating immediate explant, cold perfusion, recipient iliac vascular repair, and ultimately implantation of the graft on the contralateral side (Fig. 5).

**Fig. 6:**
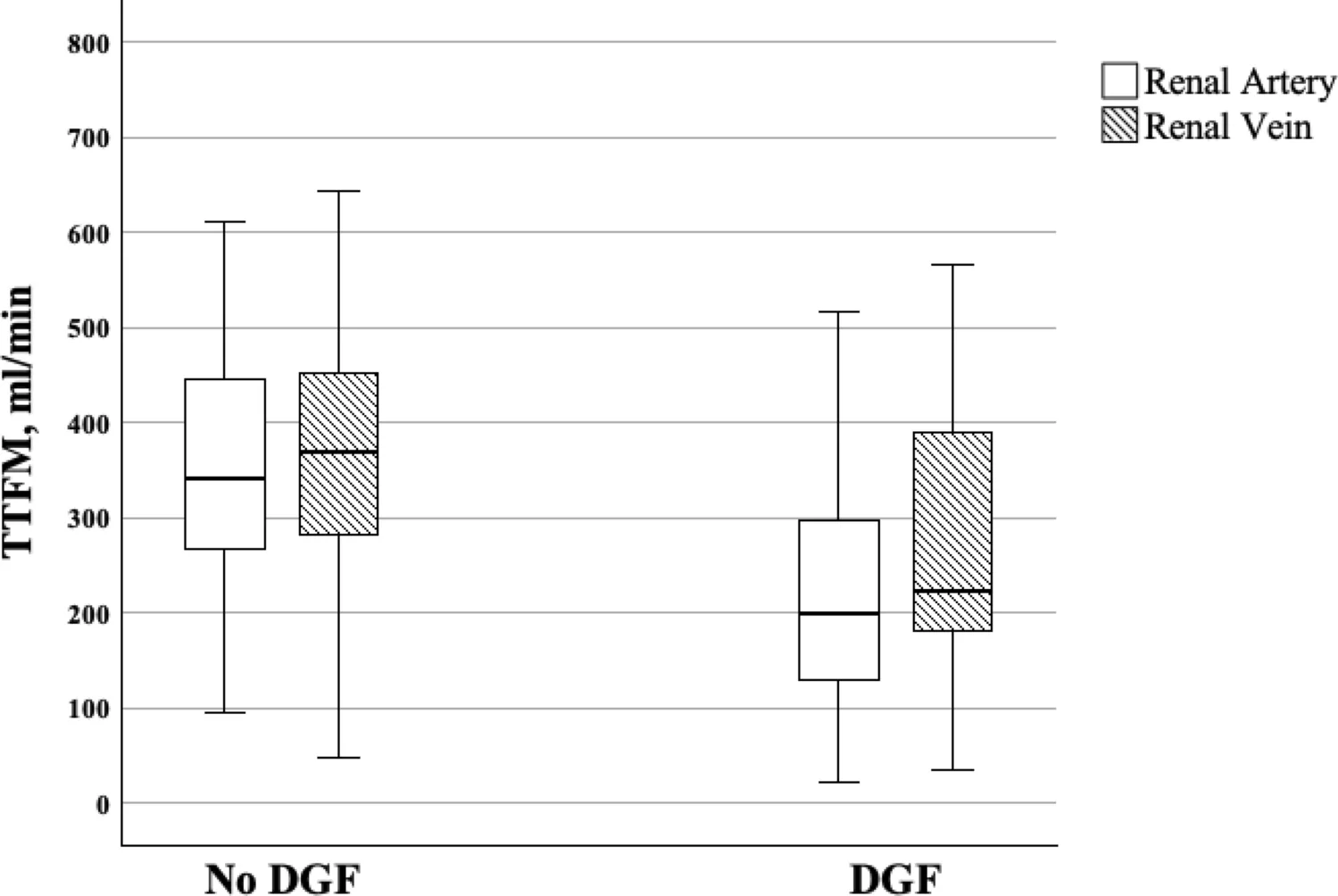
Boxplots of renal artery and vein transit time flow measurements (TTFM) according to the development or not of delayed graft function (DGF). * Renal artery TTFM in kidney transplants developing or not DGF: *p* < 0.001. § Renal vein TTFM in kidney transplants developing or not DGF: *p* = 0.015

The arterial TTFM in patients with immediate graft function was significantly higher than in patients developing DGF (341 (267–448) vs. 200 (119–298) ml/min, *p* < 0.001), as well as the renal vein TTFM (369 (272–456) vs. 222 (170–391) ml/min, *p* = 0.015), Fig. 6. The average PI values did not differ significantly between patients with immediate function and DGF (1.32 (0.90–1.86) vs. 1.60 (0.92–2.10), respectively, *p* = 0.155).

**Fig. 7:**
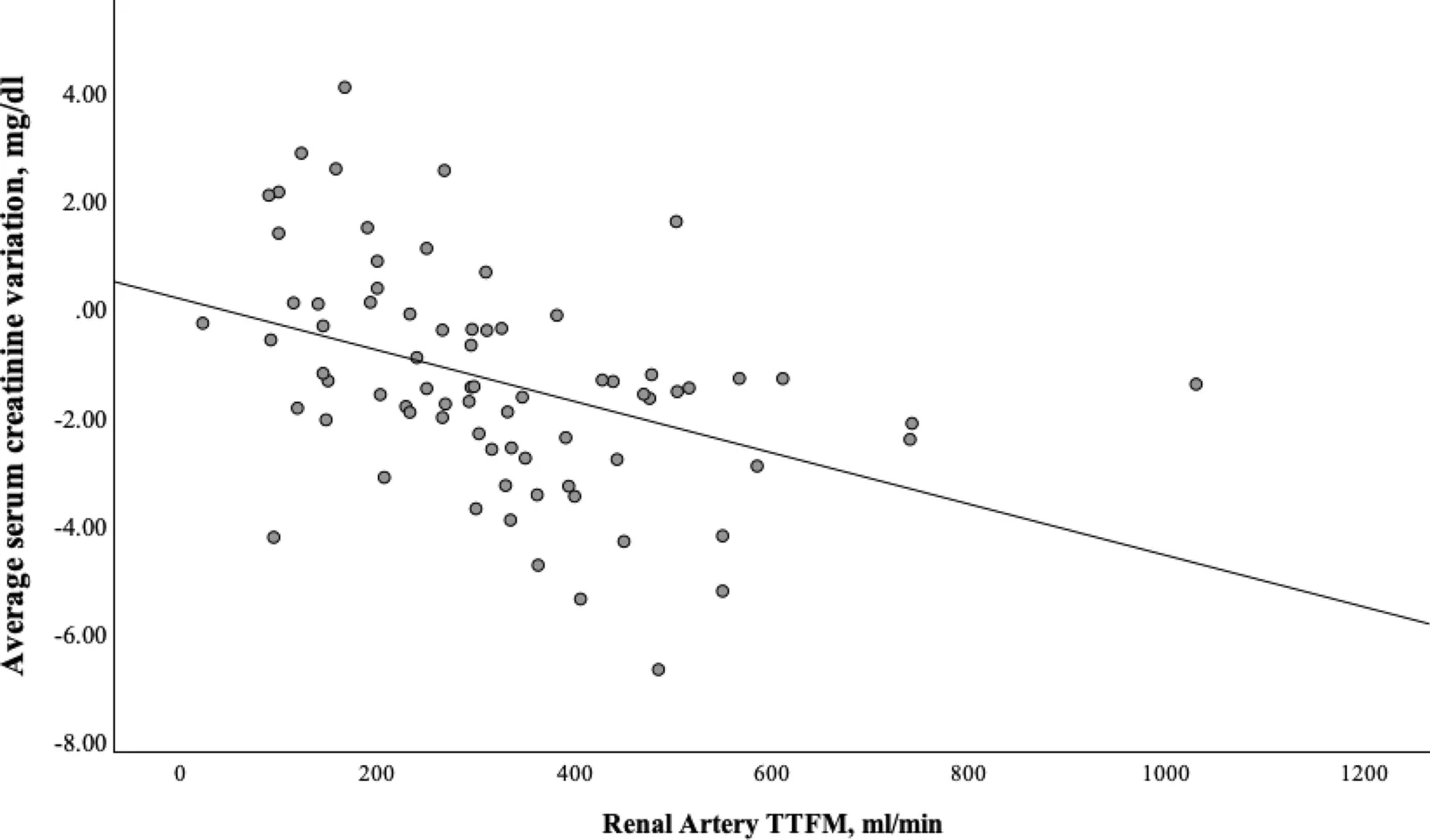
Average post-transplant serum creatinine variation (mg/dl) measured every 48-h interval (*r* = − 0.493, 95% CI − 0.652 to − 0.293, *p* < 0.001). TTFM: transit time flow measurements

Renal artery TTFM were significantly correlated to the average post KTx variation of the serum creatinine levels measured at 48 h intervals, with higher arterial TTFM corresponding to greater postoperative creatinine reductions (*r* = − 0.493, 95% CI − 0.652 to − 0.293, *p* < 0.001, Fig. 7).

**Fig. 8:**
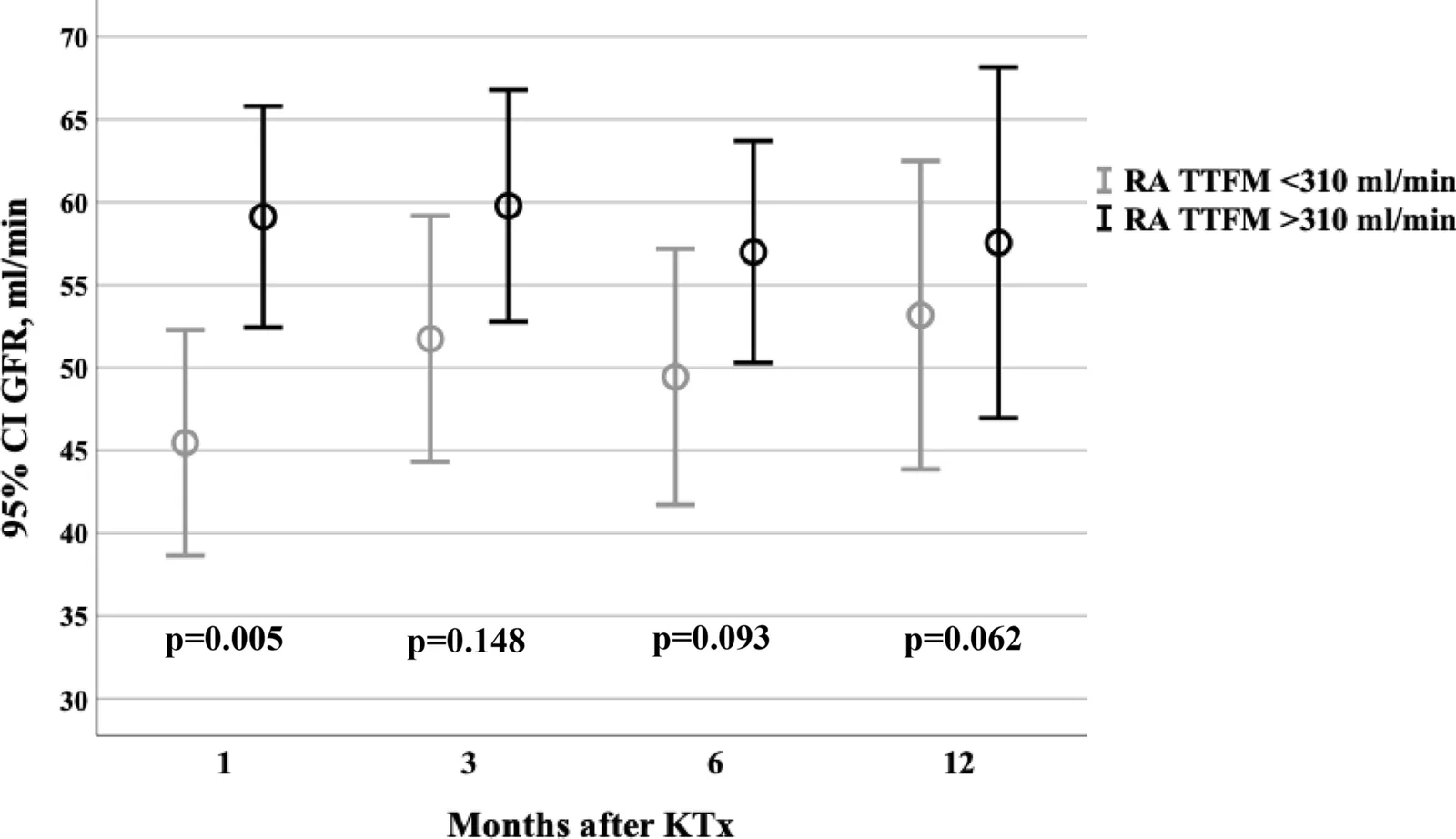
Evolution of glomerular filtration rates (GFR) after kidney transplantation (KTx) in patients with renal artery (RA) transit time flow measurements (TTFM) lower or higher than 310 ml/min

The multivariable logistic regression analysis showed the presence of donor hypertension (OR: 9.292 (2.337–36.935), *p* = 0.002) and renal artery TTFM (OR: 0.993 (0.989–0.998), *p* = 0.004) as independent risk factors for DGF development (Table 3). The identified renal artery TTFM cutoff was 310 ml/min, with a AUROC of 0.765. Patients with renal artery TTFM > 310 ml/min showed significantly higher GFR values 1 month after KTx (*p* = 0.005) and higher, although not statistically significant, GFR values 3, 6, and 12 months after KTx (*p* = 0.148, *p* = 0.093, and *p* = 0.062, respectively, Fig. 8).

**Table 3:** Multivariable analysis of risk factors for delayed graft function (DGF) development

		No DGF	DGF	*p*-Value	OR (95% CI)	*p*-Value
Donor type						
Deceased	N (%)	33 (55.9)	26 (44.1)	0.005	2.084 (0.148–29.267)	0.586
Living (reference)		15 (93.7)	1 (6.3)			
Donor hypertension						
Yes	N (%)	4 (23.5)	13 (76.5)	< 0.001	9.292 (2.337–36.935)	**0.002**
No (reference)		44 (75.9)	14 (24.1)			
Donor BMI	average ± SD	24.3 ± 3.9	26.6 ± 4.2	0.022	1.102 (0.946–1.282)	0.212
CIT, min	median (IQR)	406 (107–581)	646 (525–815)	0.001	1.002 (1.000–1.004)	0.123
Renal artery TTFM, ml/min	median (IQR)	341 (267–448)	200 (119–298)	< 0.001	0.993 (0.989–0.998)	**0.004**

OR, odds ratio; CI, confidence interval; IQR, interquartile range; BMI, body mass index; CIT, cold ischemia time; TTFM, transit time flow measurement

The p-values in bold are the statistically significant ones

## Discussion

Intraoperative TTFM in renal transplantation is an effective and reproducible assessment method that allows an immediate identification of severe vascular complications, which can lead to unfavorable outcomes and reflect graft quality and post-transplant functional recovery. Despite technical progress, vascular complications still occur in 3–15% of patients receiving KTx, negatively affecting outcomes and often necessitating invasive treatments or leading to graft loss [17–19]. Prompt recognition of graft perfusion abnormalities is of paramount importance to enable immediate intervention and resolution, thereby minimizing their potential impact.

Conventional ultrasound is a standard practice in the post transplantation setting. However, it is time-consuming, and its accuracy is highly operator-dependent for evaluating vascular flows. TTFM can represent a simple and rapid quality control method, allowing the surgeon to check the quality of the anastomoses and perform corrective actions if needed.

TTFM have been originally developed and widely adopted in cardiac surgery, proving useful in detecting intraoperative graft failures during coronary artery bypass grafting. TTFM values were shown to be associated not only with graft patency but also with postoperative clinical outcomes [20]. The principle is based on measuring the difference between the transit time upstream and downstream of an ultrasound beam. This transit time difference is directly proportional to blood volume flow, providing accurate quantification of real-time volumetric flow associated with imaging (ultrasound).

This experience has later been translated into the abdominal organ transplant setting, where TTFM arterial measurements in liver transplantation showed a correlation with graft survival, postoperative organ functional recovery, and primary nonfunction rates [21–24].

In cases of partial liver transplantation or the presence of portal hypertension, a careful check of liver graft hemodynamics and subsequent interventions can improve the imbalance between the hepatic artery and portal vein flow, eventually improving graft outcomes [23, 24].

In KTx, preliminary reports on the usefulness of TTFM assessments showed a correlation between the direct renal artery flow measurements and the anastomotic complications, as well as grafts at risk for worse outcomes [25–27].

Although ultrasound allows for the checking of peripheral perfusion of the renal cortex, it provides little information on the anastomotic sites, which can be easily assessed with TTFM. In addition, the magnified visualization of the anastomoses with high-frequency ultrasound imaging can reveal anatomic conditions and/or anastomotic defects potentially deleterious to graft function (e.g. arterial wall dissection, intimal flaps, early partial anastomotic thrombi).

Our results confirmed that the routine adoption of TTFM after graft reperfusion in KTx can effectively identify severe intraoperative vascular complications and predict the postoperative serum creatinine decrease and the occurrence of DGF, with an AUROC of 0.765. The development of DGF has been shown in a recent meta-analysis to have a detrimental effect on outcomes, with increased risks of graft failure and allograft rejection [28], and also resulting in higher costs and poorer recipient quality of life. Graft vascular TTFM were able to reflect graft quality, with higher arterial flows observed in LD recipients and higher venous flows in KTx recipients receiving SCD. This translated into better postoperative graft function, as shown by the higher GFR observed in patients with arterial TTFM > 310 ml/min (Fig. 4). These findings differed from the results of the first published series on TTFM in KTx, where graft venous flows were found to be an independent DGF predictor [26].

The PI has been previously described as a valuable parameter to detect imperfect anastomoses in coronary artery bypass grafting and flow imbalance after reperfusion in liver transplantation [22, 29]. However, according to our experience, PI proved to be a significant variable for detecting vascular complications or predicting graft outcomes.

The intraoperative identification of grafts at higher risk of DGF, in addition to its prognostication value, allows clinicians to adopt strategies aimed at reducing or avoiding other modifiable DGF risk factors, such as calcineurin inhibitors-based immunosuppression. In case of predicted high risk of DGF development, a low dose or delayed introduction of calcineurin inhibitors could be considered to prevent drug-related nephrotoxicity, which would further negatively impact graft function [30–32].

Other methods of intraoperative kidney graft perfusion assessment have been recently investigated. Laser fluorescence angiography with indocyanine green has shown promising results for the quantitative assessment of microperfusion and the DGF prediction, while O2C spectrometry has successfully detected differences in intraoperative regional tissue oxygen saturation levels in renal allografts, although the data are preliminary and based on small series [33, 34].

Performing TTFM proved to be technically simple, with no observed contraindications or complications related to the procedure. In our experience, after a brief learning curve, all operators were able to effectively measure intraoperative vascular flows in all cases without significantly prolonging the duration of surgery.

The limitations of the present study include the relatively small sample size, especially in terms of transplants of grafts after MP, and the absence of KTx performed from DCD, which do not allow a more in-depth analysis of the circulatory changes of the grafts during machine perfusion and their possible impact on post-reperfusion vascular flows.

TTFM in KTx is an effective adjunct that assists clinicians in the early identification of vascular complications, reflects graft quality, and provides reliable outcome prognostication in terms of DGF development, postoperative serum creatinine variation, and graft function. Larger series, including transplants from different types of donors and organ perfusion strategies, will help to further validate these results and better define the ideal intraoperative TTFM flow and PI value cutoffs in KTx.

## Abbreviations

ADPKD: Autosomal dominant polycystic kidney disease
AUROC: Area under the receiver operator characteristic
CIT: Cold ischemia time
DBD: Donor after brain death
DCD: Donors after circulatory death
DD: Deceased donors
DGF: Delayed graft function
IRI: Ischemia–reperfusion injury
IVC: Inferior vena cava
KDPI: Kidney donor profile index
KTx: Kidney transplants
LD: Living-donor
MP: Machine perfusion
PI: Pulsatility index
ROC: Receiver operating characteristic
SCD: Standard criteria deceased donors
TTFM: Transit time flow measurements
WIT: Warm ischemia time
